# Is the single variant form of *Plasmodium vivax* Duffy binding protein-II (PvDBP-II) adequate for inclusion in a PvDBP-II-based vaccine?

**DOI:** 10.1186/1475-2875-13-S1-P95

**Published:** 2014-09-22

**Authors:** Vahideh Valizadeh, Sedigheh Zakeri, Akram Abouei Mehrizi, Navid Dinparast Djadid

**Affiliations:** 1 Malaria and Vector Research Group (MVRG), Biotechnology Research Center (BRC), Pasteur Institute of Iran, Tehran, Iran

## 

The binding domain of Duffy protein (DBP-II) is a leading vaccine candidate of *Plasmodium vivax*. In order to develop a successful *vivax* malaria vaccine based on DBP-II, the antigenic diversity and also naturally occurring functional antibodies to different PvDBP-II variant types in the various populations must be known. To define whether the polymorphisms in PvDBP-II influenced the nature of functional antibody responses, this investigation was designed to evaluate naturally acquired antibodies to five circulating variant forms of DBP-II antigens in infected individuals with *P. vivax* living in hypoendemic areas in Iran. Sequence diversity of *pvdbp-II* gene was performed in 63 Iranian *P. vivax* isolates collected during 2008-2012. The sequencing analysis showed twenty two single nucleotide polymorphisms in PvDBP-II, resulting in 16 different haplotypes among the Iranian *P. vivax* isolates. Five of 16 genetically distinct variants were expressed in *E. coli*, and anti-DBP-II responses were measured in *P. vivax*-infected individuals (n = 202). Also, by performing immune-depletion ELISA experiments, antibody responses to the conserved sites of all five allelic forms were evaluated using the corresponding and non-corresponding patients’ sera (n = 20). ELISA results revealed that naturally acquired anti-PvDBP-II IgG were recognized all five expressed variant forms with no statistically difference (*P* > 0.05, Cochran’s Q test). The antibody depletion experiments also showed presence of the cross-reactive antibody responses to heterologous variants of PvDBP-II in Iranian individuals who were infected with distinct allelic forms of the PvDBP-II. Finally, all five examined variant forms of DBP-II were expressed transiently on the surface of COS-7 cells to determine whether people exposed to *vivax* malaria acquire antibodies that have the ability to block erythrocyte cytoadherence to PvDBP-II. The anti-DBP-II IgG block heterologous and homologous expressed DBP-II function, indicating that the protective immunity against PvDBP-II binding is not strain specific. In conclusion, the present results indicate that the single variant of PvDBP-II is adequate to be included in a PvDBP-II-based vaccine.

